# Impact of income on mental health during the COVID-19 pandemic: Based on the 2020 China family panel survey

**DOI:** 10.3389/fpsyg.2022.977609

**Published:** 2022-08-11

**Authors:** Dongliang Yang, Bingbin Hu, Zhichao Ren, Mingna Li

**Affiliations:** ^1^Northeast Asian Research Center, Jilin University, Changchun, China; ^2^Department of Regional Economics, School of Northeast Asian, Jilin University, Changchun, China; ^3^Department of Preschool Education, School of Literature, Changchun University, Changchun, China

**Keywords:** income, mental health, salary satisfaction, interpersonal relationship, COVID-19, China

## Abstract

Since December 2019, the COVID-19 has continued to rage, and epidemic prevention policies have limited contact between individuals, which may has a great influence on the income of individuals, exacerbate anxiety and depression, and cause serious mental health problems. The current study aims to examine the association between income and mental health during the COVID-19 pandemic by using the data of 9,296 observations from the 2020 China Family Panel Studies. Employing ordinary least squares regression and two-stage least squares regression, we find the significant positive effect of income on Chinese mental health during this pandemic. In addition, the number of cigarettes smoked per day has significant negative effects on mental health. Education levelˎmarriage and exercise frequency have significant positive correlation with mental health. Furthermore, the impact of income on individuals of different groups is heterogeneous during this pandemic. The impact of income for well-educated individuals is less strong than their less-educated counterparts. People who exercise regularly respond less strongly to changes in income than those who do not exercise. Finally, individuals’ salary satisfaction and interpersonal relationship are shown to be the potential mechanism for the effect of income on Chinese mental health.

## Introduction

Income has a critical impact on people’s living conditions. Many researchers exploring their impact on dietary nutrition and aspects such as vaccinations, renewable energy, attention deficit and hyperactivity disorder, physical activity, and household water consumption (Baigorri et al., 2022; Jiménez-Zazo et al., 2022; Jin et al., 2022; Mehmood et al., 2022; Valsta et al., 2022; Wang et al., 2022). All of them prove that income can affect people’s lives and physical health. Also, the personal income is closed relationship with people’s state of depression, anxiety, etc (Gou et al., 2022). Those persons with lower income are less able to access health-promoting goods and services and are difficult to maintain a feeling of control or security over their lives. Therefore, low-income people are more likely to suffer from mental disorders (Golberstein, 2015; Kiely et al., 2015; Thomson et al., 2022).

The importance of mental health is confirmed by the World Health Organization (Grad, 2002), which defines health as “the state of complete physical, mental and social well-being and not just the absence of disease or infirmity.” Mental health is closely related to individuals’ living quality and work efficiency. Many studies have shown that mental illness has great harm to individuals and society, such as domestic violence, physical illness and poor interpersonal relationships (Yanguas et al., 2011; Bradbury-Jones and Isham, 2020; Usher et al., 2020; Pelissier et al., 2021; Larsson et al., 2022). Severe mental illness can even lead to suicide (Czeisler et al., 2022). In the world, approximately 703,000 people die by suicide every year (World Health Organization, 2021a).

The novel coronavirus disease (COVID-19) was first detected in December 2019 (Zhu et al., 2022) and spread rapidly. The World Health Organization (WHO) declare it is a global pandemic in the second week of March 2020 (World Health Organization, 2021b). COVID-19 is widely disseminated, lacks specific drugs, and is life-threatening if not treated in time. In addition, the information overload of the epidemic has caused a huge impact on the public’s psychology, causing them to feel panic, anxiety and depression. In order to stop the continued spread of the COVID-19 epidemic, the government has implemented strict epidemic prevention measures and limited contact between individuals (Fang et al., 2021a), which has a great influence on individuals’ income and may exacerbate anxiety and depression.

Related research shows that the COVID-19 pandemic has had a significant negative impact on people’s mental health (Killgore et al., 2022; Zhai and Du, 2022). Most scholars’ analyses take the COVID-19 pandemic as a whole. For example, Xiong et al. (2022) reported that the COVID-19 pandemic had caused high levels of anxiety, depression, and stress in many different countries, which may have a negative impact on individual’s mental health. This result was also confirmed by Sánchez et al. (Huremović, 2019; Cao et al., 2020; Galli et al., 2022; Sánchez-Rodríguez et al., 2022). McAlearney et al. (2022) studied the mental health status of first responders (including police, firefighters, and emergency medical services) and their influencing factors during the pandemic, and many similar studies have been conducted in the literature (Cohen Veterans Network, 2020; Stogner et al., 2020; Hendrickson et al., 2022). The economic developments of all countries in the world are severely hampered by the COVID-19 pandemic (Islam, 2021; Mishra and Mishra, 2021; Nwosa, 2021; Fang et al., 2022a; Huang et al., 2022; Nawo and Njangang, 2022). Also, a large number of residents’ incomes have declined significantly during the COVID-19 pandemic (Fang et al., 2021b, 2022b; Umiński and Borowicz, 2021). The reduction of income may influent the individuals’ mental health. Ao et al. (2020) and Liang et al. (2021) examined the impact of income on mental health during the pandemic and showed that very wealthy people had the lowest levels of anxiety during the COVID-19 pandemic. In contrast, the proportion of participants showing significant depressive symptoms was significantly higher among those who are disadvantaged in economy.

Recent trends have also led to a proliferation of research on the link between human activity and mental health during the COVID-19 pandemic. For example, Quarta et al. (2022) analyzed the association of physical activity and outdoor leisure time with the mental health of college students. Foong et al. (2022) conducted a study on the relationship between Internet use and mental health in the elderly. Laurinaitytė et al. (2022) studied the association between health risk behaviors and mental health among Lithuanian university students. In addition, studies have shown that persistent loneliness and social isolation have adverse effects on the mental health of college students (Charbonnier et al., 2022).

Though the existing literature has investigated the determinants of mental health during the COVID-19 pandemic from different perspectives, little attention has been paid to the impact from the specific economic perspective, such as individual income, especially for Chinese. This article attempts to fill a gap in the relevant literature on the impact of income on mental health during the COVID-19 pandemic. We used data from the 2020 China Family Panel Study (CFPS; China Family Panel Studies, 2021), whose stratified and multi-stage sampling design enables the sample to represent almost 95% of the Chinese population, covering different ages, education levels, and national region, useful in determining the general impact of income on individual mental health. We focus on China for three reasons. First, China’s social security system is imperfect, with a series of problems such as narrow coverage, low level of security, and inadequate laws. Given this, income has a bigger impact on the Chinese. Second, many Chinese are facing serious challenges of mental health problems. The latest epidemiological survey shows that the weighted lifetime prevalence of various mental illnesses among the Chinese population over the age of 18 is 16.6% (Huang et al., 2019), and the overall mental health literacy is at a moderately low level. Third, in Chinese social life, the importance of mental health is often overlooked. In fact, mental health issues affect the quality of development in China.

This paper not only investigates the impact of income on mental health, but also considers the heterogeneous effects and mechanisms of income on mental health. Therefore, our study proposes 5 hypotheses.

Individual’s life is affected by income from different aspects (Ettner, 1996). For example, lower income can bring huge economic pressure to person’s life, which may makes individuals face more psychological crises, showing psychological symptoms such as anxiety, loneliness, and sensitivity to human relationships. On the other hand, higher income increases people’s financial ability to afford a better budget for the higher living standards and professional services for maintaining healthy lifestyles, improving factors such as depression, impaired relationships, and food security (Ao et al., 2020; Thomson et al., 2020; Xiao et al., 2020; Liang et al., 2021; Jiang et al., 2022). Based on the above analysis, we propose Hypothesis 1.

*Hypothesis* 1. Income has a significant positive effect on individual's mental health.Covid-19 had a greater impact on the mental health of less-educated participants (Sánchez-Rodríguez et al., 2022). First, well-educated people have more social and economic resources than less-educated people. They tend to have higher incomes and live without economic pressure, which is beneficial to mental health. Second, most of well-educated people have an optimistic outlook on life. It is important to cope with the difficulties in the life, including the negative effects of low incomes (Xiao et al., 2020). In sum, the impact of income on mental health may not be the same for people with different educational levels. Therefore, Hypothesis 2 arises.

*Hypothesis* 2. The impact of income for well-educated individuals is less strong than it is for their less-educated counterparts. Caponnetto et al. (2021) argue that appropriate exercise helps avoid physical and mental problems in children and adults by promoting neuronal plasticity. In addition, the existing studies indicates that physical exercise can reduce anxiety, stress and depressive symptoms (Marques et al., 2020; Loureiro et al., 2021).Those who exercised regularly were less negatively affected by low income. The effect of income on mental health may not be the same for people with different frequency of exercise. Based on the above analysis, we propose Hypothesis 3.

*Hypothesis* 3. People that exercises regularly show a less strong responses to changes of income than those who do not.There is an influence mechanism in the relationship between income and mental health. Income is an objective variable, while the mental health is come from the individual’s subjective feelings. Considering the same income, people may have completely different subjective feelings. So we take salary satisfaction as a mediation variable. Existing research shows that salary satisfaction has a positive impact on personal mental health, because people with high salary satisfaction have more active emotions and life attitudes than those with low salary satisfaction (Sorhagen and Wurster, 2017). In addition, research by Wu and Zhou (2017) also shows that higher income than one’s own expectations will lead to higher happiness. Therefore, we propose Hypothesis 4.

*Hypothesis* 4. Personal income affects mental health through its effect on salary satisfaction. Pelissier et al. (Pelissier et al., 2021; Larsson et al., 2022) point out that person who is lack of social activity is apt to the psychological stress caused by social isolation and social exclusion. During the COVID-19 pandemic, socially inactive people are at higher risk for psychological stress. Furthermore, good interpersonal relationship is the result of participation in various social activities. The high self-evaluated score of interpersonal relationships is beneficial to erase psychological distress due to the social activity. And income has a direct impact on interpersonal relationship. Research by Lee et al. (2021) shows that low-income boys are at higher risk for smartphone addiction, and that smartphone use reduces the frequency and quality of their interactions with friends. Han (2013) finding that the interpersonal interactions of students at all levels are significantly affected by their family’s economic status. Therefore, we propose Hypothesis 5.

*Hypothesis* 5. Personal income has an impact on mental health through its effect on interpersonal relationship.

## Materials and methods

### Study design

The data used in this study come from the China Family Panel Study (CFPS), a nationally representative, biannual longitudinal survey of Chinese communities, families, and individuals launched in 2010 by the Institute of Social Science Survey of Peking University, China. The CFPS is designed to studies on economic activities, education outcomes, family dynamics and relationships, migration, and health. Its stratified multi-stage sampling design enables the sample to represent almost 95% of the Chinese population, which is conducive to further heterogeneity analysis. CFPS takes individuals and families as the main research subjects at the same time. The on-site interviews use three technical tools: CAPI (computer-aided face-to-face interview), CATI (computer-aided telephone interview) and CAWI (computer-aided online interview). Face-to-face interviews are the mainstay, supplemented by internet and telephone interviews. The project will be carried out in 25 provinces/cities/autonomous regions in China (excluding Hong Kong, Macau, Taiwan, and Xinjiang Uygur Autonomous Region, Tibet Autonomous Region, Qinghai Province, Inner Mongolia Autonomous Region, Ningxia Hui Autonomous Region, and Hainan Province) in 2020. Unfortunately, for the purpose of protecting the privacy of the respondents, we cannot learn how CFPS participants were recruited and invited to the study. To examine the impact of income on mental health during the COVID-19 pandemic, this study used the 2020 baseline survey for analysis. Out of 28,590 observations, only 9,296 could be used for the current study due to concerns over missing data or uncertain answers. Ordinary least squares (OLS) and two-stage least squares (TSLS) were used for econometric analysis to investigate the impact of income on mental health.

### Statistical analysis

Statistical analysis was performed using the econometric software STATA version 16. We report the mean, standard deviation, minimum and maximum values of the variables in Table 1. Given that mental health is a continuous variable, we constructed the OLS and TSLS to investigate the causal relationship between income and mental health. In our robustness check, we used logit and probit models to estimate the effect of income on mental health. To investigate the mechanisms, we estimate the impact of natural disasters on individuals’ salary satisfaction and interpersonal relationship using both OLS and the ordered probit model. All reported *p*-values were two-tail. The level of statistical significance was set at *p* < 0.05.

**Table 1:** Descriptive statistics of the key variables.

Variable	Definition	Mean	SD	Min	Max
Mental Health	Individual’s mental health	0.035	4.071	−21.165	4.537
Income	Income for the past year(in log)	10.399	0.885	7.313	12.255
Wage	Per hour wage	2.593	0.970	−1.138	6.070
Gender	Dummy variable equals 1 if the individual is male, and 0 for female	0.592	0.492	0	1
Marital	Dummy variable equals 1 if the individual is married, and otherwise 0	0.774	0.418	0	1
Age	Individual’s age	39.432	12.040	16	83
Smoke-do or not	Dummy variable equals 1 if the individual has smoked in the past month, and otherwise 0	0.339	0.473	0	1
Smoke-number	Number of cigarettes smoked per day	4.694	8.206	0	60
Drink	Dummy variable equals 1 if the individual drank alcohol more than 3 times per week, and otherwise 0	0.149	0.356	0	1
Insurance	Individual has social insurance (1 for yes)	0.904	0.294	0	1
Medical expenses	Individual’s medical expenses	3.095	3.325	0	10
Exercise	Exercise frequency	1.550	2.157	0	7
Education Level	Highest degree completed	2.606	1.469	0	7
Salary satisfaction	Individual’s salary satisfaction	3.481	0.982	1	5
Interpersonal relationship	Relationship between popularity	6.986	1.752	0	10

The ordinary least squares method is used to estimate the impact of income on individual mental health for testing Hypothesis 1. The specific equation is as follows:


(1) $$\text{MentalHealth}=\beta_{0}+\beta_{1}\text{Income}+\sum_{1}^{j}\beta_{j}{\text{Control}}_{j}+\varepsilon$$

(2) $$\text{MentalHealth}=\beta_{0}+\beta_{1}\text{Wage}+\sum_{1}^{j}\beta_{j}{\text{Control}}_{j}+\varepsilon$$

Among them, MentalHealth represents the dependent variable (mental health index); Income represents the explanatory variable (the natural logarithm of the individual’s annual income); Wage represents the explanatory variable (hourly wages); ${\mathrm{Control}}_{j}$ is a series of other explanatory variables, includes dummy variables for gender, marital status, smoking, drinking, medical insurance, and continuous variables for age, number of cigarettes smoked daily, medical expenses, exercise times weekly and education level, and the random error term is determined by ε express.

Since the above model may ignore the biases caused by reverse causality, this study uses the instrumental variable method to further examine the impact of income on individual mental health. According to the study of Yang and Li (2019), the two-stage least squares method was used to obtain unbiased parameter estimates, and then a consistent estimate of the effect of endogenous variables on the explained variables was obtained. The specific equation is as follows:

First stage:


(3) $$Y=\beta_{1}+\beta_{2}\text{Fund}+\sum_{1}^{j}\beta_{j}{\text{Control}}_{j}+\varepsilon$$

Second stage:


(4) $$\text{MentalHealth}=\beta_{1}+\beta_{2}Y^{\prime }+\sum_{1}^{j}\beta_{j}{\text{Control}}_{j}+\varepsilon$$

In Equation (3), Y is the dependent variable (the natural logarithm of the individual’s annual income or per hour wage), and Fund is the instrumental variable (whether to provide housing provident funds). In Equation (4), $Y^{\prime }$ is the predicted value in the first stage, ${\mathrm{Control}}_{j}$ is the control variable, includes dummy variables for gender, marital status, smoking, drinking, medical insurance, and continuous variables for age, number of cigarettes smoked daily, medical expenses, exercise times weekly and education level, and ε is the residual item.

Finally, we drive a mediation effect test based on the mediating effect model (Sobel, 1987) and analyze whether income affects individual mental health through salary satisfaction and interpersonal relationship for testing Hypothesis 4 and 5. The specific equation is as follows:


(5) $$\text{MentalHelath}=\beta_{0}+\beta_{2}\text{X}+\sum_{1}^{j}\beta_{j}{\text{Control}}_{j}+\varepsilon_{1}$$

(6) $$M=\gamma_{0}+\gamma_{1}X+\sum_{1}^{j}\lambda_{j}{\text{Control}}_{j}+\varepsilon_{2}$$

(7) $$\text{MentalHealth}=\delta_{0}+\delta_{1}X+\delta_{1}M+\sum_{1}^{j}\delta_{j}{\text{Control}}_{j}+\varepsilon_{3}$$

In Equation (5), *X* is the independent variable (person’s annual income or hourly wage); in Equation (6), *M* is the mediator variable (salary satisfaction or interpersonal relationship). ${\mathrm{Control}}_{j}$ is the control variable, includes dummy variables for gender, marital status, smoking, drinking, medical insurance, and continuous variables for age, number of cigarettes smoked daily, medical expenses, exercise times weekly and education level. Equation (5)–(7) are used to estimate the total, allocation and mediation effect of income on individual mental health.

### Measures

#### Dependent variable: Mental health

The dependent variable in this paper is the mental health status of individuals in China. In order to evaluate individual’s mental health more objectively and comprehensively, following existing studies (Zhang et al., 2022), the mental health index is derived from the 6-item short form of the Center for Epidemiologic Studies of Depression (CES-D) in the CFPS. (CES-D questions: 1. In the past week, how many times have you felt down? 2. In the past week, how many times did you feel that you were struggling to do anything? 3. In the past week, how many times have you had poor sleep? 4. In the past week, how many times have you felt lonely? 5. In the past week, how many times have you felt sad? 6. In the past week, how many times have you felt that life could not go on? Individuals were asked to indicate the frequency of their feelings on a four-scale metric— “Almost never (less than a day),” “Sometimes (1–2 days),” “Often (3–4 days),” “Most of the time (5–7 days).” These responses are assigned a value of 4 to 1, respectively).

The Center for Epidemiological Studies Depression Scale (CES-D) is a self-rating tool developed by the National Institutes of Mental Health (Radloff) in 1977 to evaluate the current level of depression in community populations (Radloff, 1977). This scale has been translated and used in many countries and has good reliability and validity (Van de Velde et al., 2011). We used SPSS26 to calculate the reliability of the questionnaire, and the Cronbach’s alpha coefficient for the CES-D scale was 0.763, which showed that the CES-D scale has good reliability.

Referring to the method of Cheng et al. (Kling et al., 2007; Chen and Wu, 2021), the personal mental health index is an equally weighted sum of the standardization of score variable (z-score) of the above 6 components. The specific equation is as follows:


(8) $${\text{MentalHealth}}_{i}=\sum_{j=1}^{6}\frac{x_{ij}-u_{j}}{\sigma_{j}}\;$$

In the equation, ${\mathrm{MentalHealth}}_{i}$ represents the mental health index of the individual, $x_{ij}$ represents the individual data of each variable, $u_{j}$ and $\sigma_{j}$ represent the overall mean and standard deviation of the variable, respectively. The mental health index calculated from this is −21.165 to 4.537. The higher the mental health index, the better the individual’s mental health, and vice versa.

#### Independent variable: Income

We use two measures of income as independent variables. The first variable is the individual’s annual income (Income), which is calculated as the sum of the after-tax wage income from the main job and general job in the past 12 months. The second variable is hourly wage (Wage). It measures how much an individual earns per hour worked. In order to obtain regression results with economic implications, income and wage were processed by taking the natural logarithm.

#### Mediating variable

In order to study the mechanism by which income affects individual mental health, we selected two variables: salary satisfaction and interpersonal relationship. Salary satisfaction ranges from 1–5, with 1 being the least satisfied and 5 being the most satisfied. Interpersonal relationship ranges from 0 to 10, with 0 representing the worst relationship and 10 representing the best relationship.

#### Control variables

In order to better address the problem of selection bias caused by omitted variables, this study selects as many control variables as possible that affect both personal income and mental health. Mainly include the following control variables: dummy variables for gender, marital status, smoking, drinking, medical insurance, and continuous variables for age, number of cigarettes smoked daily, medical expenses, frequency of exercise and education level. Among them, smoke-do or not means have you smoked in the past month? 1 means yes, 0 means no; smoke-number indicates how many cigarettes you currently smoke on average per day; drink indicates whether you drank more than 3 times a week in the past month. 1 means yes, 0 means no; the frequency of physical activity reflects how often you have participated in physical fitness and leisure activities in the past 12 months. Ranges from 0 to 7 (never participates = 0; average less than 1 time per month = 1; average more than 1 time per month but less than 1 time per week = 2; 1–2 times per week =3; 3–4 times per week on average = 4; 5 and more times per week on average = 5; 1 time per day = 6; 2 times per day or more = 7). Education level ranges from 0 to 7 (illiteracy = 0; primary school = 1; junior high school = 2; high school/secondary school/technical school/vocational high school = 3; junior college = 4; undergraduate = 5; master = 6; Ph.D. = 7).

#### Instrumental variable

Appropriate instrumental variables need to be strongly correlated with endogenous explanatory variables and not correlated with disturbance terms. Therefore, exogenous policy intervention or exogenous interference to some samples is a better choice as an instrumental variable. Existing research shows (Kling et al., 2007; Jin, 2018; Zhan et al., 2018; Lu and Wan, 2020) that housing provident funds have a significant impact on income, which is exogenous relative to mental health. That is to say, the housing provident fund has an impact on residents’ mental health by affecting changes in income, and endogenous variables are the only way for the housing provident fund to ultimately affect the dependent variable. Therefore, the housing provident fund is determined as the instrumental variable of this research. In the CFPS questionnaire, “whether to provide housing provident fund” was selected as the measure of housing provident fund.

### Participants characteristics

The descriptive statistics of the variables used in this paper are shown in Table 1. It can be seen that the average age of individuals in the sampled sample is 39.63 years old, and 59.2 percent of them are male. The mental health index of the sample ranges from −21.16 to 4.537, and the insured sample accounts for 90.45% of the total sample.

## Empirical results

### Baseline results

When investigating the causal relationship between income and mental health, a person’s smoking and drinking behaviors are usually highly correlated, and the high correlation between variables raises concerns about multicollinearity, which may lead to larger biases. We use the variable inflation factor (VIF) to check for multicollinearity in our model. Table 2 reports the VIF of each variable. In each case, the VIF was less than the empirical value of 10, indicating that multicollinearity was not a major problem.

**Table 2:** The variance inflation factor of each variable.

Variables	VIF	VIF
Income	1.26	
Wage		1.24
Gender	1.61	1.58
Marital	1.25	1.24
Age	1.49	1.48
Smoke-do or not	3.25	3.25
Smoke-number	2.87	2.87
Drink	1.15	1.15
Insurance	1.03	1.03
Medical expenses	1.01	1.01
Exercise	1.10	1.11
Education Level	1.49	1.52
Mean VIF	1.59	1.59

VIF represents variable inflation factor.

Table 3 reports baseline results regarding the impact of income on individual mental health. Columns (1) and (3) include only the individual’s annual income and hourly wages, respectively. Columns (2) and (4) contain a set of control variables that affect individual’s mental health. The effects of columns (1) and (3) show that increase in income have a significant positive effect on individual mental health. When controlling for a set of covariates in columns (2) and (4), results from the OLS model showed that income are a significant predictor of individual mental health, showing a positive correlation. These results validate Hypothesis 1.

**Table 3:** OLS results of the effects of income on mental health.

	(1)	(2)	(3)	(4)
Income	0.554^***^ (11.71)	0.286^***^ (5.51)		
Wage			0.536^***^ (12.42)	0.281^***^ (6.00)
Gender		0.749^***^ (7.10)		0.777^***^ (7.44)
Marital		0.897^***^ (8.21)		0.902^***^ (8.28)
Age		0.008 (1.85)		0.006 (1.36)
Smoke-do or not		−0.109 (−0.70)		−0.111 (−0.71)
Smoke-number		−0.026^**^ (−3.10)		−0.026^**^ (−3.05)
Drink		0.099 (0.80)		0.098 (0.80)
Insurance		0.295^*^ (2.09)		0.286^*^ (2.03)
Medical expenses		−0.203^***^ (−16.40)		−0.202^***^ (−16.34)
Exercise		0.087^***^ (4.37)		0.082^***^ (4.12)
Education Level		0.237^***^ (6.97)		0.226^***^ (6.62)
_cons	−5.728^***^ (−11.59)	−4.622^***^ (−8.47)	−1.356^***^ (−11.35)	−2.280^***^ (−9.63)
*N*	9,296	9,296	9,296	9,296
*R* ^2^	0.015	0.065	0.016	0.066
Adj. *R*^2^	0.014	0.064	0.016	0.065

*t* statistics in parentheses.

* *p* < 0.05;

** *p* < 0.01;

*** *p* < 0.001.

As for the control variables, gender is shown as a positive number in columns (2) and (4). This suggests that men’s mental health is better than women’s. The education Level regression coefficient is positive, indicating that education has a positive effect on mental health. The marital status coefficient was positive and statistically significant. The results showed that married adults had higher mental health than unmarried adults. The coefficient of exercise frequency is positive, indicating that regular physical exercise has a positive impact on individual mental health. The number of cigarettes smoked per day and medical expenses had significant negative effects on mental health.

### Endogeneity

In the regression results of the first stage in Table 4, the instrumental variables have a significant positive effect on the core explanatory variables, indicating that there is a strong correlation between the instrumental variables and the endogenous variables. The *F* values of the first-stage regression results were 276.99 and 210.55, both greater than 10, indicating that there is no weak instrumental variable phenomenon in the two-stage model, and the housing provident fund is a good instrumental variable; In addition, the Hausman endogeneity test was used, and the Hausman results passed the 1% statistical level test, indicating that individual annual income and hourly wages as endogenous explanatory variables have endogeneity problems, and instrumental variables need to be used for unbiased estimation. From the results of the second stage, it can be seen that after using instrumental variables to adjust the estimation bias, the impact of personal annual income and hourly wage on mental health is still positive and significant, regression coefficients up to 0.672 and 1.386. Comparing the results of OLS regression with the results of two-stage regression, it can be seen that there is a significant gap between the estimation results of the instrumental variable method and the estimation results of OLS, there is a large bias in the OLS estimation.

**Table 4:** Two-stage least squares regression results.

	One stage regression	Second stage regression	One stage regression	Second stage regression
Income		0.672^**^ (2.92)		
Wage				1.386^**^ (2.85)
Gender	0.383^***^ (18.88)	0.606^***^ (4.51)	0.283^***^ (12.37)	0.471^**^ (2.73)
Marital	0.268^***^ (12.68)	0.791^***^ (6.30)	0.259^***^ (10.84)	0.612^***^ (3.62)
Age	−0.006^***^ (−7.74)	0.011^*^ (2.38)	0.000 (0.12)	0.006 (1.50)
Smoke-do or not	0.028 (0.91)	−0.122 (−0.78)	0.036 (1.05)	−0.153 (−0.95)
Smoke-number	0.004^*^ (2.23)	−0.027^**^ (−3.20)	0.001 (0.80)	−0.027^**^ (−3.07)
Drink	0.071^**^ (2.96)	0.070 (0.56)	0.076^**^ (2.81)	0.012 (0.09)
Insurance	0.118^***^ (4.28)	0.254 (1.77)	0.145^***^ (4.68)	0.132 (0.82)
Medical expenses	−0.004 (−1.67)	−0.200^***^ (−16.04)	−0.008^**^ (−2.96)	−0.192^***^ (−14.21)
Exercise	0.006 (1.52)	0.083^***^ (4.13)	0.026^***^ (5.83)	0.051^*^ (2.09)
Education level	0.181^***^ (27.94)	0.152^*^ (2.53)	0.242^***^ (33.04)	−0.062 (−0.47)
Housing provident fund	0.068^***^ (22.33)		0.033^***^ (9.58)	
_cons	9.652^***^ (216.69)	−8.324^***^ (−3.76)	1.439^***^ (28.60)	−3.832^***^ (−5.31)
*N*	9,296	9,296	9,296	9,296
*R* ^2^	0.247	0.060	0.200	0.010
Adj. *R*^2^	0.246	0.059	0.199	0.009

*t* statistics in parentheses.

* *p* < 0.05;

** *p* < 0.01;

*** *p* < 0.001.

### Robustness check

In the robustness test, we apply the logit model and the probit model to estimate the impact of income on individual mental health. To do this, we substitute a dummy variable for the individual’s mental health. This false value takes 1 if the mental health value is greater than the mean of mental health index, and 0 otherwise. Columns (1), (3), and (5) in Table 5 report the estimated results of the income on mental health measured by dummy variable under the conditions of OLS, logit model and probit model, respectively. Columns (2), (4) and (6) report the results of wage. According to the results, the personal income and wage coefficients are both positive and statistically significant. That is, increased income has a positive effect on mental health. All in all, the results in Table 5 are basically consistent with those in Table 3.

**Table 5:** Robustness test results.

	(1)	(2)	(3)	(4)	(5)	(6)
	OLS	OLS	Logit	Logit	Probit	Probit
Income	0.027^***^ (4.17)		0.112^***^ (4.16)		0.070^***^ (4.18)	
Wage		0.029^***^ (5.05)		0.123^***^ (5.03)		0.123^***^ (5.03)
Control variable	YES	YES	YES	YES	YES	YES
_cons	0.081 (1.21)	0.295^***^ (10.13)	−1.779^***^ (−6.23)	−0.875^***^ (−7.05)	−1.102^***^ (−6.24)	−0.875^***^ (−7.05)
*N*	9,296	9,296	9,296	9,296	9,296	9,296
*R* ^2^	0.044	0.045				
Adj. *R*^2^	0.043	0.044				

*t* statistics in parentheses.

*** *p* < 0.001.

### Heterogeneity

To better understand the relationship between income and mental health, we examine the heterogeneity of effects by dividing the sample into different levels of education and frequency of exercise.

In order to test Hypothesis 2, the research samples were divided into Less-educated group and Well-educated group (If the education level reaches 3 or above, it is Well-educated, otherwise it is Less-educated). Table 6 presents the results of the heterogeneous effect of income on mental health at different levels of education. The results of the study show that people with lower education levels show a stronger response to changes in income, regardless of whether the study is based on individual annual income or hourly wage income. These results validate Hypothesis 2.

**Table 6:** Heterogeneous effects of different education levels on income.

	(1)	(2)	(3)	(4)
	Less-educated	Well-educated	Less-educated	Well-educated
Income	0.447^***^ (6.27)	0.200^**^ (2.63)		
Wage			0.389^***^ (5.96)	0.232^***^ (3.40)
_cons	−6.376^***^ (−8.14)	−2.334^**^ (−2.83)	−2.606^***^ (−8.12)	−0.822^*^ (−2.50)
*N*	4,942	4,354	4,942	4,354
*R* ^2^	0.075	0.042	0.074	0.043
Adj. *R*^2^	0.073	0.040	0.072	0.041

*t* statistics in parentheses.

* *p* < 0.05;

** *p* < 0.01;

*** *p* < 0.001.

To test Hypothesis 3, we included a dummy variable to measure exercise frequency. The dummy equals 0 if the individual never participates in physical activity, and otherwise 1. Table 7 presents the results of the heterogeneous effect of income on mental health at different exercise frequencies. The results of the study showed that people who did not exercise were more responsive to changes in income than those who exercised regularly, regardless of individual annual income or hourly wage as the research object. The results in Table 7 validate Hypothesis 3.

**Table 7:** Heterogeneous effects of different exercise frequencies on income.

	(1)	(2)	(2)	(3)
	No exercise	Regular exercise	No exercise	Regular exercise
Income	0.383^***^ (5.30)	0.153^*^ (1.97)		
Wage			0.337^***^ (5.13)	0.205^**^ (2.85)
_cons	−5.982^***^ (−7.84)	−2.549^**^ (−3.15)	−2.767^***^ (−8.60)	−1.380^***^ (−3.58)
*N*	5,534	3,762	5,534	3,762
*R* ^2^	0.068	0.052	0.068	0.053
Adj. *R*^2^	0.066	0.049	0.066	0.050

*t* statistics in parentheses.

* *p* < 0.05;

** *p* < 0.01;

*** *p* < 0.001.

### Mechanisms

In order to explore the mechanism by which income affects mental health, this section uses the Causal Steps Approach to analyze whether income affects individual mental health through the relationship between salary satisfaction and interpersonal relationship.

To test Hypothesis 4, we estimated the effect of individual annual earnings, hourly wage on salary satisfaction, and salary satisfaction on mental health by OLS. Given that salary satisfaction is recorded on an ordinal scale, we also employed an ordered probit model to investigate the impact of personal income on mental health. Columns (1)–(4) of Table 8 report the effect of individual’s annual income and hourly wage on salary satisfaction, and columns (5) and (6) report the effect of salary satisfaction on mental health. As can be seen from Table 8, positive and statistically significant coefficients are shown. This suggests that income has an impact on individual’s mental health through its effect on salary satisfaction. The results in Table 8 validate Hypothesis 4.

**Table 8:** With salary satisfaction as a mediating variable.

Salary satisfaction					MentalHealth	
	(1)	(2)	(3)	(4)	(5)	(6)
Variable	OLS	OLS	Ordered probit	Ordered probit	OLS	OLS
Income	0.183^***^ (14.36)		0.194^***^ (13.58)		0.202^***^ (3.89)	
Wage		0.144^***^ (12.50)		0.155^***^ (12.03)		0.215^***^ (4.59)
Salary satisfaction					0.456^***^ (10.87)	0.455^***^ (10.89)
Control variable	YES	YES	YES	YES	YES	YES
_cons	1.521^***^ (11.33)	3.071^***^ (52.58)			−5.315^***^ (−9.73)	−3.678^***^ (−13.72)
*N*	9,296	9,296	9,296	9,296	9,296	9,296

*t* statistics in parentheses.

*** *p* < 0.001.

To test Hypothesis 5, we estimated the effect of personal annual income, hourly wage on interpersonal relationship, and the effect of interpersonal relationship on mental health by OLS. Given that interpersonal relationship is recorded on an ordinal scale, we also employ an ordered probit model to investigate the effect of income on interpersonal relationship. Columns (1)–(4) in Table 9 report the effect of individual’s annual income and hourly wage on interpersonal relationship, and columns (5) and (6) report the effect of interpersonal relationship on mental health. As can be seen from Table 9, positive and statistically significant coefficients are shown. This suggests that income has an impact on mental health through its impact on interpersonal relationship. The results in Table 9 validate Hypothesis 5.

**Table 9:** With interpersonal relationship as a mediating variable.

Interpersonal relationship					MentalHealth	
	(1)	(2)	(3)	(4)	(5)	(6)
Variable	OLS	OLS	Ordered probit	Ordered probit	OLS	OLS
Income	0.084^***^ (3.68)		0.044^**^ (3.23)		0.262^***^ (5.10)	
Wage		0.078^***^ (3.76)		0.042^***^ (3.40)		0.259^***^ (5.58)
Interpersonal relationship					0.276^***^ (11.82)	0.275^***^ (11.80)
Control variable	5.272^***^ (21.87)	5.969^***^ (57.07)			−6.076^***^ (−10.94)	−3.923^***^ (−14.36)
_cons	9,296	9,296	9,296	9,296	9,296	9,296

*t* statistics in parentheses.

** *p* < 0.01;

*** *p* < 0.001.

## Discussion

Since the outbreak of the COVID-19 pandemic in 2019, its influence has attracted a lot of attention and research. Many studies are related to the mental health that we study in this article. Compared with the current study, the novelty of this paper is four-fold. First, previous studies focused on specific populations, such as first responders, college students, the elderly, and were not nationally representative. Our research investigates the general impact of personal income on individual’s mental health. Second, we select housing provident fund as an instrumental variable to solve the possible endogeneity problem. Third, we examine the heterogeneity of effects by dividing the sample into different education levels and frequency of exercise. These results help us understand the heterogeneity of the effects of personal income on mental health. Finally, existing research fail to provide mechanisms on how income affects mental health. This paper reveals that income can affect mental health through the impact on salary satisfaction and interpersonal relationship.

One feature of current research is tending to focus on specific populations and regions. For example, Brasso et al. (2022) conducted a meta-analysis of the existing literature and found that the COVID-19 pandemic has produced a significant impact on the mental health of young people. Rapisarda et al. (2022) analyzed mental health symptoms of mental health workers in Lombardy and Quebec during COVID-19. However, the above studies are based on descriptive studies and lack empirical support. Our study employs ordinary least squares and two-stage least squares (TSLS) to investigate the causal effects of income on individual mental health and to provide empirical evidence. Baseline results were consistent with previous studies (Sánchez-Rodríguez et al., 2022). Furthermore, most studies used non-probability samples. Unlike Pais-Ribeiro et al. (Pais-Ribeiro et al., 2022), our study is derived from large-scale micro survey data. Also we provide a mechanistic analysis. Different from the previous studies that the effect of income appears to be mediated largely by debt (Jenkins et al., 2008), our study identifies the mechanism variables are salary satisfaction and interpersonal relationship.

Another feature of current research is apt to prove that there is a positive correlation between income and mental health. For instant, most scholars (Ao et al., 2020; Thomson et al., 2020; Liang et al., 2021) believe that income has a positive impact on mental health during COVID-19. However, due to the obvious endogeneity between income and mental health, it is difficult to obtain consistent estimates by OLS. Obviously, there is a bidirectional causal relationship between income and mental health. Chisholm et al. (2016) found that higher income improves mental health, which can further improve livelihoods or economic security by increasing productivity, promoting labor market participation, and increasing the income. This study attempts to solve this problem by selecting the housing provident fund as an instrumental variable. The empirical results report the causal effect of personal income on mental health and test the hypotheses. Furthermore, little discuss is on the heterogeneity of income’s effect on individual mental health. Our research shows that income affects individuals’ mental health differently, depending on the individual’s level of education and frequency of exercise. That is, less education showed a stronger response to changes in income, regardless of whether they were based on annual personal income or hourly wages. People who did not exercise were more responsive to changes in income than those who exercised regularly.

However, this paper has limitations in some aspects. First, the sample data is cross-sectional. We can only estimate the short-term effects of income on individual’s mental health, not taking into account the long-term effects. Also the conclusions that can be drawn from our survey results only involve absolute income, we cannot analyze whether income loss occurred during the epidemic and the impact of income loss on individual mental health. Second, although we try our best to include factors that may affect individual’s mental health, the model cannot include all external factors that affect individual’s mental health. Third, aside from data limitations, we measure mental health in a very general way. An interesting avenue of future research can be predicted regarding the long-term and dynamic effects of income on individual mental health. In addition, studies can investigate the causal relationship between income and specific psychological problems, such as postpartum depression.

Several policy implications can be derived from this analysis. First, our research shows that income has a significant positive effect on an individual’s mental health. Therefore, governments and society as a whole may need to provide assistance to low-income people. This help should be directed not only to financial subsidies, but also to effective mental health care. Particular attention should be paid to those who are less educated and do not exercise regularly. Finally, the government should also develop mental health services for vulnerable groups and encourage social workers to conduct home visits. Additionally, programs involving outdoor activities can be provided to encourage low-income groups to get out of their homes and start interacting with others, while maintaining appropriate social distancing to prevent the spread of COVID-19, improve their relationships, and reduce their depression.

## Conclusion

Given the importance of mental health in daily life, there has been an increasing amount of research on this topic during the COVID-19 pandemic. This paper uses 9,296 items of data from the 2020 CFPS survey to investigate the causal relationship between income and mental health. One of the most important findings to emerge from the paper is that income has a positive effect on mental health during the pandemic.

The effect of heterogeneity was further analyzed by segmenting the sample according to education level and exercise. On the one hand, the findings show that changes in income have a slightly stronger effect on people with less education than those with well education. On the other hand, those who exercised regularly showed a less strong responses to changes in income than those who do no. Our study also investigates the mechanisms by which income affects mental health, showing that income affects mental health through its effects on salary satisfaction and interpersonal relationship.

## Data availability statement

The original contributions presented in the study are included in the article/supplementary material, further inquiries can be directed to the corresponding author.

## Author contributions

DY conceived and designed the research, provided guidance throughout the entire research process, and responsible for all R&R works. BH and ZR participated in data analysis. BH and ML wrote the article. All authors contributed to the article and approved the submitted version.

## Funding

This research was funded by the National Social Science Fund of China (Grant number: 21AJL006), Northeast Revitalization Program of Jilin University (Grant number: 22dbzx13), Labor relations Program of Jilin University (Grant number: 2021LD011), Special Fund of Jilin University (Grant number: YSZ202109), and Adolescent Development Program of Jilin Province (Grant number: 2022jqy-zj2).

## Acknowledgments

The authors would like to express sincere gratitude to Geoffrey Hewings from University of Illinois at Urbana-Champaign and the peer reviewers for their valuable suggestions.

## Conflict of interest

The authors declare that the research was conducted in the absence of any commercial or financial relationships that could be construed as a potential conflict of interest.

## Publisher’s note

All claims expressed in this article are solely those of the authors and do not necessarily represent those of their affiliated organizations, or those of the publisher, the editors and the reviewers. Any product that may be evaluated in this article, or claim that may be made by its manufacturer, is not guaranteed or endorsed by the publisher.
